# Unraveling Ductal Carcinoma In Situ in an Unscreened Population: Understanding Its Natural History Through Advanced Presentation and Management

**DOI:** 10.7759/cureus.80950

**Published:** 2025-03-21

**Authors:** Quratulain Ali, Rabia Niaz, Rufina Soomro

**Affiliations:** 1 Department of Breast Surgery, Liaquat National Hospital and Medical College, Karachi, PAK

**Keywords:** breast cancer, delayed presentation, ductal carcinoma in situ, risk stratification, sentinel lymph node biopsy, unscreened population

## Abstract

Background: Ductal carcinoma in situ (DCIS) is a non-invasive breast cancer with debated management strategies, particularly in unscreened populations where delayed detection often leads to advanced presentations. Understanding DCIS in this context is crucial for improving risk stratification, treatment, and outcomes.

Objective: This study aims to explore the clinicopathologic features, progression, and outcomes of DCIS in an unscreened population, comparing findings with national and international studies.

Methods: We conducted a retrospective analysis of 172 patients diagnosed with isolated DCIS at Liaquat National Hospital and Medical College, Karachi, Pakistan, from January 2019 to December 2023. Data collected included demographics, presenting symptoms, imaging findings, biopsy methods, histopathologic features, and treatment details. Statistical analysis was performed using SPSS Statistics for Windows, Version 25 (Released 2017; SPSS Inc., Chicago, United States), with p ≤ 0.05 considered significant.

Results: Of 4690 breast cancer cases, 3.6% were isolated DCIS. The median age was 51 years, with 66% postmenopausal. The most common symptom was a palpable lump (68%), with only 3.5% detected via screening. High-grade DCIS was prevalent (41.7%), with comedo necrosis in 23.7%. Tumor size exceeded 5 cm in 25.8% of cases. Breast-conserving surgery (BCS) was performed in 39.9% of patients, with a 15.4% re-surgery rate. Mastectomy and sentinel lymph node biopsy were required in 56.6% of cases. The upgrade rate to invasive carcinoma was 39.9%, higher than global averages. Estrogen receptor positivity was noted in 70.9% of patients.

Conclusion: DCIS in unscreened populations presents more aggressively, with larger, higher-grade tumors and a significant risk of progression to invasive disease. The findings emphasize the need for targeted screening and tailored management strategies to improve outcomes. Future research should focus on optimizing diagnostic and therapeutic approaches in such high-risk groups.

## Introduction

Ductal carcinoma in situ (DCIS), although a non-invasive disease, poses a complex challenge in management. While DCIS remains confined within the basement membrane of the ductal system, its biologic nature and optimal management remain debated, both in screened populations due to concern of overtreatment and in unscreened populations due to under-diagnosis and advanced presentations [1].

DCIS constitutes 15-20% of all diagnosed breast cancer cases in countries practicing regular screening protocols [2]. Out of these, 90% are screen-detected [3]. In unscreened populations, however, patients present with symptoms such as a palpable lump or thickening, nipple discharge, or ulceration of the nipple in almost 74% of cases [4].

Isolated DCIS in unscreened populations sheds light on the clinicopathologic features, progression, and outcomes of this precursor lesion. It gives an opportunity to explore the natural history of the disease and understanding DCIS, in such contexts, is imperative for effective risk stratification, progression to invasive disease, personalized treatment approaches, and informed decision-making regarding surgical interventions and adjuvant therapies [1].

This article aims to critically examine the demographics, clinical presentation, pathological features, molecular subtypes, upgrade to invasive disease as well as management strategies for breast and axilla within unscreened populations and to compare presentation, progression, and management outcomes with national and international data. By evaluating the implications of late-stage detection with symptomatic presentations, we aim to enhance our understanding of the disease spectrum, optimize diagnostic approaches, refine risk assessment models, and tailor management strategies to ensure optimal outcomes for affected individuals.

## Materials and methods

Study design

We conducted a retrospective analysis of medical records for patients diagnosed with isolated DCIS at Liaquat National Hospital and Medical College, Karachi, Pakistan. The study spanned from January 2019 to December 2023 and focused on patients aged 28-78 years. The analysis aimed to evaluate clinicopathological factors in comparison to unscreened cases exploring the natural history, management strategies in such advanced presentations, and adherence to established guidelines in such complex cases.

Inclusion and exclusion criteria

The inclusion criteria for the study involved female patients aged 28-78 years who were diagnosed with isolated DCIS on initial biopsy and presented at Liaquat National Hospital and Medical College during the specified study period. Patients with concurrent invasive carcinoma or microinvasion and metastatic axillary lymph nodes on initial workup were excluded from the analysis.

Data collection

Data was extracted from the hospital’s medical records files. This included demographics such as age, menopausal status, use of hormonal therapy (HRT) or oral contraceptives, and family history of breast cancer. Clinical presentation details were recorded, including presenting complaints and imaging findings. Biopsy information was documented, focusing on the mode of biopsy and findings from the initial biopsy. Histopathology results included tumor size, grade and type of DCIS, presence of comedo necrosis, and hormone receptor status. Treatment details were also collected, covering the type of surgery performed, axillary management, adjuvant hormone therapy, radiation therapy, and surgical tumor-free margins, where complete excision was defined as ≥2 mm. If complete excision was not achieved, re-excision or mastectomy with sentinel lymph node (SLN) biopsy was performed according to the National Comprehensive Cancer Network (NCCN) guidelines. SLN biopsy was performed alongside breast-conserving surgery (BCS) in the primary setting for patients with indications such as a large palpable lump, high-grade DCIS on biopsy, and financial constraints preventing re-surgery. We followed the established guidelines for axillary clearance, in which axillary clearance is recommended in cases where macrometastases are identified in one or more SLNs for patients undergoing mastectomy, and in more than two SLNs for those undergoing BCS.

Statistical analysis

Descriptive statistical analysis was employed to classify the results using the SPSS Statistics for Windows, Version 25 (Released 2017; SPSS Inc., Chicago, United States). Frequencies and percentages were computed for the qualitative variables. Quantitative variables presented as mean ± SD and chi-square test/Fisher's exact test used for findings association. P-value ≤ 0.05 will be considered as significant.

## Results

Out of 4690 patients diagnosed with breast carcinoma at our hospital over the period of five years, 172 (3.6%) were identified as isolated DCIS. The median age of patients was 51 years (±12.5) and 114 (66.3%) were postmenopausal. Only two (1.2%) patients had a history of hormonal replacement therapy use. The most common presentation was breast lump (117, 68%) followed by nipple discharge with no lump (32, 18.6%). Nipple ulcers were less common, seen in five (2.9%) of the patients, and only six (3.5%) were detected in asymptomatic patients by screening mammogram. The family history of breast carcinoma was positive in 14 (8.1%) patients.

Radiologically, mammography detected hyperdense lesions as the most prevalent found in 69 (62.7%) patients. Microcalcifications were present in 24 (21.8%) and architectural distortions were less common, seen in seven (6.4%) of patients. Ultrasound findings were non-specific, showing heterogeneous areas in 34 (29.8%), and hypoechoic lesions were observed in 23 (20.2%) of patients.

DCIS was diagnosed in 90 (53.3%) patients by tru-cut biopsy and in 48 (27.9%) patients via image-guided biopsy, including ultrasound-guided tru-cut biopsy (26.0%), stereotactic biopsy (0.6%), and wire-localized excision biopsy (1.8%). Excision was done in 14 (8.3%) of patients while wedge biopsy was the mode of biopsy in three (1.8%) of patients (Table 1).

**Table 1:** Clinicopathological characteristics of DCIS in an unscreened population (N=172) SD: Standard deviation; DCIS: Ductal carcinoma in situ; ER: Estrogen receptor

Variable	N (%)
Age (years); mean ± SD	51.70 ± 12.50
Age Groups	
<50 years	72 (41.9)
≥50 years	100 (58.1)
Grade (n=127)	
Low	29 (22.8)
Intermediate	45 (35.4)
High	53 (41.7)
Tumor Size (n=143)	
<1 cm	30 (21.0)
1-2 cm	8 (5.6)
2.1-5.0 cm	60 (42.0)
5.1-8.0 cm	37 (25.9)
>8 cm	8 (5.6)
Comedo Necrosis (n=131)	
Present	31 (23.7)
Absent	100 (76.3)
Menopausal History	
Pre-menopausal	58 (33.7)
Post-menopausal	114 (66.3)
Hormonal Therapy	
Yes	2 (1.2)
No	170 (98.8)
Family History	
Yes	14 (8.1)
No	158 (91.9)
Clinical Presentation	
Lump	117 (68.0)
Nipple discharge	32 (18.6)
Paget’s disease/nipple ulcers	5 (2.9)
Screening mammogram	6 (3.5)
Others	12 (7.0)
Mode of Biopsy (n=169)	
Excision biopsy	14 (8.3)
Microdochectomy	10 (5.9)
Needle localization biopsy	3 (1.8)
Stereotactic biopsy	1 (0.6)
Tru-cut biopsy	90 (53.3)
Ultrasound-guided biopsy	44 (26.0)
Wedge biopsy	3 (1.8)
Other	4 (2.4)
Mammographic Findings (n=110)	
Architectural distortion	7 (6.4)
Hyperdense lesion	69 (62.7)
Microcalcifications	24 (21.8)
Other	10 (9.1)
Ultrasound Findings (n=114)	
Cystic area	10 (8.8)
Dilated ducts	22 (19.3)
Heterogeneous area	34 (29.8)
Hyperdense lesion	22 (19.3)
Hypoechoic lesion	23 (20.2)
Nipple thickening	1 (0.9)
Normal	2 (1.8)
Hormone Receptor Status (n=141)	
ER Positive	100 (70.9)
ER Negative	41 (29.1)
Re-Surgery (n=143)	
Yes	22 (15.4)
No	121 (84.6)
Re-Surgery Type (n=22)	
Margin re-excision	9 (40.9)
Mastectomy	11 (50.0)
Sentinel lymph node biopsy (SLNB)	2 (9.1)
Axillary Status (n=143)	
Positive	20 (11.6)
Negative	123 (86.0)
Histopathology Findings (n=143)	
Benign	4 (2.8)
Microinvasive	24 (16.8)
Invasive	33 (23.1)
DCIS	82 (57.3)

**Figure 1:**
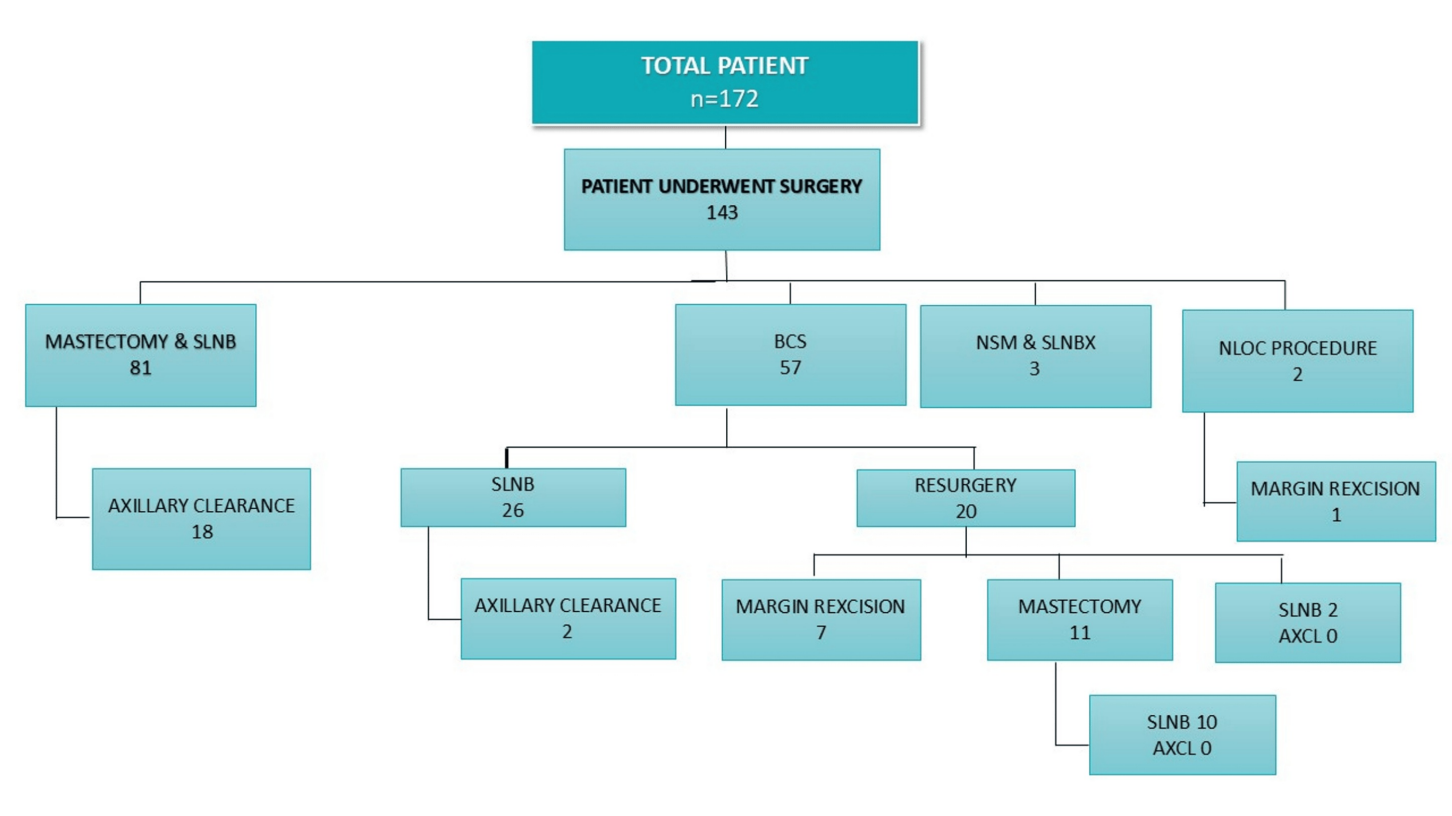
Surgical management of DCIS in an unscreened population SLNB: Sentinel lymph node biopsy, NSM: Nipple-sparing mastectomy, NLOC: Needle localization, BCS: Breast-conserving surgery, AXCL: Axillary clearance

Out of 143 patients who underwent surgery, mastectomy with SLN biopsy was the most common procedure, performed in 81 patients (56.6%), followed by BCS in 57 patients (39.9%), which included oncoplastic techniques such as the Grisotti flap. SLN biopsy was performed on a total of 122 patients (85.3%), including those who underwent mastectomy and BCS. Among BCS patients, 38 (26.5%) underwent SLN biopsy, and axillary clearance was performed in 20 patients (16.4%). In the BCS group (n=57), 21 patients (14.6%) required re-surgery, including 11 mastectomies, nine margin re-excisions, and two SLN biopsies. Additionally, nipple-sparing mastectomy with breast reconstruction was performed for three patients (Figure 1).

The histopathology of the final specimen revealed isolated DCIS including mucinous differentiation, encapsulated papillary carcinoma, and solid papillary carcinoma in 82 (57.3%) of cases, while 33 (23.1%) had invasive disease and 24 (16.8%) had microinvasion. Tumor size was ≤2 cm in 38 cases (26.4%), between 2 and 5 cm in 60 cases (42%), and ≥5 cm in 45 cases (31.3%), ranging from small foci to a maximum of 9 cm.

Almost half of the patients had high-grade DCIS (53, 41.7%), with comedo necrosis observed in 31 (23.7%) cases. Paget’s disease was noted as an associated pathology in seven (4.1%) of cases. Other associated pathologies included intraductal papilloma in 13 patients (7.6%) and lobular carcinoma in situ in two patients (1.2%). The most common DCIS subtypes were solid (15.1%) and cribriform (14%). An upgrade in final histopathology in 57 (39.9%) of patients was noted, in which 33 (23.8%) were invasive cancer and 24 (16.1%) showed only microinvasion. Estrogen receptor (ER) positive status was found in 100 (70.9%) of the patients (Table 1).

Table 2 highlights a significant correlation between clinicopathological factors and histopathological findings. High-grade tumors are likely to be invasive, whereas low-grade tumors are commonly associated with DCIS (p=0.017). Larger tumors (≥2.1cm) show a higher risk of invasion compared to smaller ones (p=0.007). Axillary node involvement is significantly linked to invasive disease (p<0.001). Other factors, including comedo necrosis, menopausal status, and imaging findings, show no significant correlation with disease upgrade. These results emphasize the role of tumor size, grade, and biopsy method association with advanced cases.

**Table 2:** Frequency and association of histopathology findings in our population (n=143) DCIS: Ductal carcinoma in situ; ER: Estrogen receptor A chi-square test was applied. ↕ indicates Fisher's exact test was applied. * indicates p-value < 0.05 considered as significant.

	Histopathology Findings N (%)			Test Statistics	P-value
	Microinvasive	Invasive	DCIS		
Grade (n=126)					
Low	3 (13)	4 (12.9)	21 (29.2)	12.048	0.017*
Intermediate	9 (39.1)	18 (58.1)	18 (25)		
High	11 (47.8)	9 (29)	33 (45.8)		
Tumor Size ↕					
<1 cm	3 (12.5)	0 (0)	27 (31.4)	26.454	<0.001*
1-2 cm	1 (4.2)	2 (6.1)	5 (5.8)		
2.1-5.0 cm	7 (29.2)	22 (66.7)	31 (36)		
5.1-8.0 cm	10 (41.7)	7 (21.2)	20 (23.3)		
>8 cm	3 (12.5)	2 (6.1)	3 (3.5)		
Comedo Necrosis (n=130)					
Present	6 (26.1)	9 (30)	16 (20.8)	1.088	0.580
Absent	17 (73.9)	21 (70)	61 (79.2)		
Menopausal History					
Pre	9 (37.5)	10 (30.3)	25 (29.1)	0.630	0.764
Post	15 (62.5)	23 (69.7)	61 (70.9)		
Hormonal Therapy ↕					
Yes	1 (4.2)	1 (3.0)	0 (0)	3.828	0.203
No	23 (95.8)	32 (97)	86 (100)		
Clinical Presentation ↕					
Lump	18 (75)	29 (87.9)	56 (65.1)	6.163	0.577
Nipple discharge	4 (16.7)	3 (9.1)	17 (19.8)		
Pagets disease/nipple ulcers	1 (4.2)	0 (0)	3 (3.5)		
Screening Mammogram	0 (0)	0 (0)	3 (3.5)		
Others	1 (4.2)	1 (4.2)	7 (8.1)		
Mode of Biopsy (n=141) ↕					
Excision biopsy	0 (0)	0 (0)	9 (10.5)	20.694	0.042*
Microdiscectomy	3 (13)	1 (3.1)	5 (5.8)		
Needle loc biopsy	1 (4.3)	0 (0)	0 (0)		
Stereotactic	0 (0)	0 (0)	1 (1.2)		
Trucut biopsy	13 (56.5)	26 (81.3)	39 (45.3)		
Ultrasound-guided	5 (21.7)	5 (15.6)	26 (30.2)		
Wedge biopsy	0 (0)	0 (0)	3 (3.5)		
Other	1 (4.3)	0 (0)	3 (3.5)		
Mammographic Findings (n=100) ↕					
Architectural distortion	0 (0)	2 (7.7)	4 (7.1)	13.658	0.018*
Hyperdense lesion	6 (33.3)	19 (73.1)	38 (67.9)		
Microcalcification	10 (55.6)	3 (11.5)	9 (16.1)		
Other	2 (11.1)	2 (7.1)	5 (8.9)		
Ultrasound Finding (n=102) ↕					
Cystic area	1 (5.9)	1 (3.6)	5 (8.8)	15.590	0.144
Dilated ducts	1 (5.9)	2 (7.1)	12 (21.1)		
Heterogenous area	10 (58.8)	10 (35.7)	12 (21.1)		
Hyperdense lesion	4 (23.5)	8 (28.6)	10 (17.5)		
Hypoechoic lesion	1 (5.9)	6 (21.4)	16 (28.1)		
Nipple thickening	0 (0)	0 (0)	1 (1.8)		
Normal	0 (0)	1 (3.6)	1 (1.8)		
Hormone Receptor Status (n=131)					
ER Positive	15 (62.5)	20 (69)	59 (75.6)	1.707	0.426
ER Negative	9 (37.5)	9 (31)	19 (24.4)		
Re-Surgery ↕					
Yes	4 (16.7)	3 (9.1)	15 (17.4)	1.262	0.549
No	20 (83.3)	30 (90.9)	71 (82.6)		
Axillary Status ↕					
Positive	6 (25)	11 (33.3)	3 (3.5)	20.595	<0.001*
Negative	18 (75)	22 (66.7)	83 (96.5)		

## Discussion

The early detection of DCIS through mammographic screening has been extensively debated, particularly concerning the overtreatment of non-progressive lesions. In our study, only 3.5% of cases were detected asymptomatically, reflecting a global trend from the pre-screening era, where rates were below 5%, and emphasizing the impact of limited screening in Pakistan, where the incidence remains low [5], while the incidence increased up to 20% with regular screening protocols internationally. In previous studies from Pakistan, the incidence as low as 1.1% has been reported due to a lack of screening [6]. This low percentage is consistent with studies from populations (3.1%) in Johannesburg with limited access to screening programs, where many cases of DCIS are still diagnosed at later stages due to delayed detection [4].

The younger age distribution suggests a potential need for targeted awareness and screening programs in young women who are not typically the focus of standard screening guidelines. Similar results have been seen in other studies comparing screened and non-screened women diagnosed with DCIS (40.4% versus 38.2% were aged 50-54 years, 31.0% versus 30.4% were aged 55-59 years, and 28.6% versus 31.4% were aged 60-64 years) [7].

Globally, mammographic screening has been shown to reduce the mortality rate of breast cancer, partly by identifying early-stage lesions such as DCIS, which have a high potential for progression to invasive disease [8]. However, the benefits of early detection can be nuanced. In our study, the majority of patients (68%) with DCIS presented with a breast lump, which is consistent with findings in unscreened populations (23/42) of South Africa where DCIS is often diagnosed at a palpable stage [9]. Studies have shown that unscreened populations tend to have larger tumors at the time of diagnosis, and their tumors are more likely to exhibit higher grades and comedo necrosis [4,9], similar to results from our study. A study from the Netherlands showed no significant difference in the grade distribution between the screen-detected DCIS and the non-screen-detected DCIS (16.4-18.8% were low grade, 27.2-31.6% were intermediate grade, and 52.0-54.0% were high grade) [10].

The treatment of DCIS differs significantly between screened and unscreened populations, largely due to differences in the stage of presentation. In screened populations, BCS is commonly performed, as it provides excellent cosmetic outcomes and is associated with similar survival rates to mastectomy in many cases of early-stage DCIS. Our study reported a high mastectomy rate (56.6%) compared to Western cohorts, reflecting the aggressive nature of DCIS cases in unscreened populations. The need for re-surgery in 15.4% of BCS cases further underscores the challenges of managing advanced presentations. Comparatively, studies from Johannesburg report even lower BCS rates due to the unsuitability of this approach in advanced cases [4].

Our data also underscore the importance of SLN assessment, especially in high-risk cases, with axillary clearance performed in a significant number of patients. The study reinforces current practices and emphasizes the need for precision in assessing nodal involvement. Bellver et al. performed SLN biopsy in 70% of cases with 7.2% nodal involvement from three Spanish hospitals. High chances of nodal involvement were seen with lymphovascular involvement and mastectomy [11], while 4.9% node positivity was reported in another study which is very low in comparison to our population [12].

In our cohort, the adjuvant therapy rates (radiation and HRT) were consistent with current guidelines, as 70.9% of patients had ER-positive disease, which is the most common subtype of DCIS. This aligns with studies that have shown that ER-positive DCIS is associated with a favorable prognosis, especially when treated with adjuvant therapy.

Perhaps the most significant difference between screened and unscreened populations is the risk of progression from DCIS to invasive carcinoma The upgrade rate to invasive carcinoma (39.9%) was notably higher than the 15-25% range commonly reported [12]. Factors contributing to this include larger tumor size, high nuclear grade, ER-negative status, and delayed diagnosis leading to disease progression. This finding aligns with meta-analyses that identify similar risk factors for invasive progression [12].

The limitations of the study primarily include its retrospective design, which relies on medical records and may introduce selection bias. Another limitation is that it is a single-center study, including only 172 cases over five years, which limits the generalizability of the findings to larger populations. A significant limitation is the loss of 29 patients to follow-up, which could have impacted the analysis of treatment outcomes and disease progression. There are also limitations in the details on axillary pathological nodal status as the data was retrospective and it did not support the primary goal of the study. Additionally, the study also lacks follow-up data which is not gathered. Analyzing the long-term outcomes of these patient will help strengthen the management strategies, particularly for those presenting with advanced disease.

## Conclusions

This study presents a single institution's experience of DCIS management in an unscreened population. The diagnosis of isolated DCIS is rare in our setting, and the majority of patients present with more advanced, symptomatic disease. Patients with large tumors and high-grade lesions had a higher likelihood of upgrade on final histopathology. Mastectomy rates remain higher compared to patients diagnosed through screening which can be attributed to advanced presentation, financial constraints limiting re-surgery, and limited access to radiotherapy. SLN biopsy is performed with BCS in most of our patients due to high-risk features favoring an upgrade to invasive disease. This leads to a higher number of redundant SLN biopsies, creating a management dilemma in selecting patients for SLN biopsy due to the lack of established guidelines for patients who present with advanced disease, unlike in the screened population.

We believe that DCIS requires a multidisciplinary approach and patient-tailored therapy, particularly in patients with progressive disease. The complexity of managing DCIS in unscreened populations, where late-stage detection and aggressive disease features are common, underscores the need for improved strategies. These findings advocate enhanced awareness, targeted screening programs including younger age groups, and improved management strategies. Future research should focus on refining diagnostic and therapeutic approaches to reduce the burden of advanced breast cancer in underserved regions.
